# Neuroprotective action of α-Klotho against LPS-activated glia conditioned medium in primary neuronal culture

**DOI:** 10.1038/s41598-022-21132-4

**Published:** 2022-11-07

**Authors:** Vinicius Wanatable Nakao, Caio Henrique Yokowama Mazucanti, Larissa de Sá Lima, Paloma Segura de Mello, Natacha Medeiros de Souza Port’s, Paula Fernanda Kinoshita, Jacqueline Alves Leite, Elisa Mitiko Kawamoto, Cristoforo Scavone

**Affiliations:** 1 Department of Pharmacology, Institute of Biomedical Science ICB-1, University of São Paulo, Avenida Professor Lineu Prestes, 1524, São Paulo, 05508-900 Brazil; 2 Department of Pharmacology, Institute of Biomedical Sciences, University Federal of Goias, Goiana, Brazil; 3 Laboratory of Clinical Investigation, National Institute on Aging (NIA), Bethesda, USA

**Keywords:** Biochemistry, Neuroscience

## Abstract

The α-Klotho is an anti-aging protein that, when overexpressed, extends the life span in humans and mice. It has an anti-inflammatory and protective action on renal cells by inhibiting NF-κB activation and production of inflammatory cytokines in response to TNF-α. Furthermore, studies have shown the neuroprotective effect of α-Klotho against neuroinflammation on different conditions, such as aging, animal models of neurodegenerative diseases, and ischemic brain injury. This work aimed to evaluate the effects of α-Klotho protein on primary glial cell culture against the proinflammatory challenge with LPS and how this could interfere with neuronal health. Cortical mixed glial cells and purified astrocytes were pretreated with α- α-Klotho and stimulated with LPS followed by TNFα, IL-1β, IL-6, IFN-γ levels, and NF-κB activity analysis. Conditioned medium from cortical mixed glia culture treated with LPS (glia conditioned medium (GCM) was used to induce neuronal death of primary cortical neuronal culture and evaluate if GCM-KL (medium from glia culture pretreated α-Klotho followed by LPS stimulation) or GCM + LPS in the presence of KL can reverse the effect. LPS treatment in glial cells induced an increase in proinflammatory mediators such as TNF-α, IL-1β, IL-6, and IFN-γ, and activation of astrocyte NF-κB. GCM treated-cortical neuronal culture induced a concentration-dependent neuronal death. Pretreatment with α-Klotho decreased TNF-α and IL-6 production, reverted NF-κB activation, and decreased neuronal death induced by GCM. In addition, KL incubation together with GCM + LPS completely reverts the neuronal toxicity induced by low concentration of GCM-LPS. These data suggest an anti-inflammatory and neuroprotective effect of α-Klotho protein in the CNS. This work demonstrated the therapeutic potential of α-Klotho in pathological processes which involves a neuroinflammatory component.

## Introduction

α-Klotho protein gene was discovered randomly in 1997 by Kuro-o and colleagues^1^. The α-Klotho protein can be found in two forms, a transmembrane and a soluble one, the latter composed of both the cleaved α-Klotho and the secreted α-Klotho acting in the central nervous system (CNS)^1–3^. The transmembrane α-Klotho has a short intracellular domain, and two extracellular domains, known as KL1 and KL2, and the secreted α-Klotho is a result of alternative splicing composed of only KL1^1^. These extracellular domains can be cleaved by the proteases disintegrin A and metalloproteinase 10 (ADAM10), disintegrin A and metalloproteinase 17 (ADAM17), and β-secretase 1 (BACE1). These cleavage fragments are present in the blood, urine, and cerebrospinal fluid, acting as a humoral factor^4–6^.

Evidence suggests that α-Klotho expression is predominantly circumscribed to the kidneys and the CNS^1^. This protein could be found in the choroid plexus^7^, neurons, oligodendrocytes, cortical layers, hippocampal formation^8^, and Purkinje cells^9^. The membrane-bound α-Klotho complexes with several fibroblast growth factor (FGF) receptors isoforms^1^, modulating kidney phosphate reabsorption and 1,25-dihydroxycholicalciferol (vitamin D) production for systemic regulation of phosphate homeostasis^10,11^. The soluble forms of α-Klotho act as a humoral factor and can be found in extracellular fluids such as blood, urine, and cerebral spinal fluid (CSF)^4,12,13^.

Physiological and pathological processes influence the expression of α-Klotho. Rats with spontaneous hypertension, 5/6 nephrectomized, and type 1 diabetes had their α-Klotho mRNA levels decreased^14^, and endogenous factors such as insulin and glutamate modulate α-Klotho expression in mouse neurons^15^. The α-Klotho’s expression increases significantly after birth and adulthood^2,8^, and there is a decrease during aging^16–18^.

In aging, there is a low-grade chronic systemic inflammation called inflammaging^19^. Deregulation of inflammation in the brain is associated not only with cognitive deficits related to aging^20^ but also with the pathogenesis and progression of neurodegenerative diseases^21^.

Lipopolysaccharides (LPS)-treated glial cells (a model of neuroinflammation) activates pathways involving TLR4 and the nuclear transcription factor kappa B (NF-kB). The NF-κB activity plays an important role in modulating proteins and cytokines^22^. This nuclear factor is constitutively expressed in the cytoplasm, which is bound to the inhibitor κB (IκB) protein which masks its nuclear localization signal, thus retaining it in the cytoplasm^23^. Cytokines and other pro-inflammatory mediators are involved in hippocampal neuronal functions^24^, but they can also cause damage to hippocampal working memory consolidation and LTP^24–26^. The present study investigated the role of α-Klotho and the activity of NF-kB in glial cells challenged with LPS and determined the ability of this protein to revert the neurotoxicity on neuronal culture cells caused by the GCM.

## Materials and Methods

### Chemicals and kits

Cell culture reagents were purchased from Thermo Fisher Scientific (Waltham, MA, U.S.A.). Protein assay kit was purchased from Bio-Rad (Hercules, CA, U.S.A.). TNF-α, IL-10, and IL-1β immunoassay kits were purchased from eBioscience (San Diego, CA, U.S.A.). The kits were used according to the manufacturer´s instructions. Routine reagents and LPS from *Escherichia* c*oli* (O111:B4, L2630) were purchased from Sigma Chemicals (St. Louis, MO, U.S.A.), and recombinant α-Klotho from R&D Systems (1819-KL-050, Minnesota, MN, U.S.A.). All solutions were prepared immediately before use.

### Primary cell culture

Primary mixed cortical glia culture and cortical neuron culture were prepared from C57BL/6J mice obtained at the Biomedical Sciences Institute of the University of São Paulo, São Paulo, São Paulo State, Brazil. The newborn animals were euthanized by decapitation, and cortical tissue was removed and used for both cell culture preparation (glial cells and neurons). The procedure was conducted following ARRIVE guidelines (https://arriveguidelines.org) which are similar to the Ethical Principle in Animal Research adopted by the Brazilian College of Animal Experimentation (CONCEA) and were approved by the Ethical Committee for Animal Research (CEEA) of the Biomedical Sciences Institute of the Universidade de São Paulo, São Paulo, São Paulo State, Brazil. The protocol was registered under number 12/2016 CEEA of animals used for experimentation. All methods were performed in accordance with the relevant guidelines and regulations of the standard Scientific Reports editorial criteria.

Primary glial cells were prepared as previously described^22^. Briefly, cortex from newborn C57BL/6J mice (postnatal days 1–4) was dissected in ice-cold Hanks’ balanced salt solution (HBSS) under a microscope and their meninges were removed. Small pieces of cortices were incubated in a trypsin solution (GIBCO) for dissociation at 37 °C for 20 min. DMEM (complemented with glutamine, 10% Hyclone FetalLOne III serum, GE Healthcare, and 1% Penicillin/ Streptomycin) was added to the solution to inhibit trypsin action, and cells were dissociated with a Pasteur pipette. The cells were passed through Cell Strainer, and cells were counted in a Neubauer chamber, and each Flask T75 (Sarstedt) was plated with 1 × 10^6^ cells. The medium was changed every three days and kept for 10–14 days. The culture was plated in a 6-well plate or 24-well plate for experiments. The culture used for the experiments was considered an astroglial-enriched culture based on previous data showing 91,2% astrocytes labeled with GFAP^22^. To obtain astrocyte-pure cell cultures (> 98%) for NF-kB experiments, flasks were incubated in an orbital shaker at 37 °C, 180 r/min, for 15 h as previously described^15^.

The primary cortical neuronal culture was prepared from both male and female postnatal (P1-3) mice C57BL/6 (Mus musculus). The meninges were separated from the brain, and the cortex was cut into small pieces and incubated in trypsin solution (2 mg/mL) for 20 min in a 37 °C, 5% CO_2_ incubator. After removal of trypsin solution, tissue was washed twice with HBSS. Tissue was dissociated in HBSS containing 0.1 mg/mL DNAse by mechanical trituration with a glass pipette. Cells were counted and plated (1 × 10^6^ cells) in polyethylene (Sigma-Aldrich) pre-coated dishes. Neurons were maintained for two weeks in a Neurobasal medium (GIBCO) supplement with B27 (GIBCO), 2 mM L-glutamine, 100 U/mL penicillin, 100 mg/mL streptomycin, and 0.25 mg/mL amphotericin B.

### α-Klotho and LPS treatment

On the 15th day, the cells were treated with only DMEM without fetal bovine serum (FBS), LPS, α-Klotho (AA 35–982), or LPS + α-Klotho for 24 h. A dose–response of recombinant α-Klotho and LPS treatments were made with different concentrations (α-Klotho 0.1–4.0 nM) and LPS (0.01–100 μg/mL) for 24 h to observe cell viability. After this first screening, only α-Klotho concentrations (0.1–2.0 nM) were tested at different time points (1, 4, and 24 h) to evaluate the α-Klotho effect in LPS (1 µg/mL/8 h)-induced changes in TNF-α levels. Based on these data, the concentration of 1 nM of α-Klotho and 1 µg/mL to LPS for 4 and 8 h was used to evaluate changes in NF-kB activity and IL-1β, IL-6, and IFN-γ levels, respectively.

For the experiments involving Glial conditioned Medium (GCM), on the 15th day, cell culture was pretreated with DMEM without FBS in the absence (control) or the presence of α-Klotho (1 nM) for 24 h and then challenged with 1 µg/mL LPS for 8 h. The media were collected and named GCM or GCM-KL. Primary neuronal culture cells were challenged on the 10th day by changing the normal conditioned medium by 25% or 50% of GCM or GCM-KL for 24 h. The neuronal cultures were also challenged on the 10th day by changing the normal conditioned medium by 25% or 50% of GCM (challenged with 1 µg/mL LPS for 8 h) in the absence (control) or the presence of α-Klotho (1 nM).

### Cell viability by LDH and MTT

Cell viability was estimated by Cytotox 96 non-radioactive assay (Promega). The assay was performed according to the Manufacturer's instructions. Lactate dehydrogenase (LDH) release was assayed after 24 h of treatment with α-Klotho or LPS by removing 50 μl supernatant from each well into a 96-well plate and incubating with Cytotox 96 reagents for 30 min covered with aluminum foil at room temperature. The stop solution was added, and the absorbance was measured at 490 nm. The percentage of LDH activity was measured by the ratio: (Absorbance of the sample/Absorbance of maximum activity) × 100%.

The cells were incubated with filtered MTT in DMEM without FBS at 37 °C for 2 h and 30 min. The supernatant was removed from the plate, and DMSO was added. The new supernatant was plated on a 96-well plate and measured at 570 nm. MTT % related to control was calculated by the ratio: (absorbance of the sample–absorbance of DMSO)/ (absorbance of the control–absorbance of DMSO) × 100%.

### Multiplex analysis of cytokines and chemokines

Concentrations of TNF-α, IL-1β, IL-6, IL-18, and INF-γ were simultaneously measured in 25 μL of medium from homogenized cells using a Milliplex M.A.P. kit Mice Cytokine/Chemokine Magnetic Bead Panel (Millipore, Billerica, MA) by following manufacturer’s instructions. Antibody immobilized beads were detected on a Luminex 100 xMAP technology machine (Austin, TX). Standard curves were generated for each cytokine/chemokine using standards included in the kit for serum samples. The median fluorescence intensity for each analyte was calculated using a five-point logistic parameter curve and normalized to the amount of protein in each sample.

### Immunofluorescence

To evaluate the effects of α-Klotho on NF-κB activation by LPS, after purification, astrocytes were treated for 24 h with vehicle (control) (PBS) or 1 nM α-Klotho. Inflammatory stimulation with LPS 1 μg/ml was then performed and the cells were fixed with methanol (10 min) 4 h later and washed with PBS three times for 5 min. The fixed cells were incubated with serum blocking (5% normal donkey serum in triton X-100 0.01%) for one hour and incubated overnight with primary antibodies GFAP (1:300) (3670; Cell Signaling, and RelA (p65 (1:100) (ab7970; ABCAM, USA). The primary antibody was removed, and the plate was washed with serum blocking three times for 10 min. The cells were incubated with secondary antibody (rabbit or mouse anti-donkey- Alexa 594 or 488, Thermo Fischer Scientific, 1:1000), diluted in PBS with Triton X-100 0.01% for 2 h, and protected from the light. The coverslips were washed five times with PBS for 5 min and incubated with DAPI (4'-6-Diamidino-2-phenylindole; Sigma) for 1 min in dilution 1:10.000. Slices were transferred to glass slides and analyzed in Fluorescence microscope Nikon Eclipse 80i (Nikon, Tokyo, Japan) with a camera system, Nikon Digital Camera DXM 1200C.

### Protein extraction—nucleus and cytoplasm

Culture media were removed from the 6-well plate, and the cells were scraped in cold PBS with 0.5 mM PMSF and centrifuged at 4 °C for 2 min at 13,000*g*. Pellet was resuspended in lysis buffer (10 mM HEPES pH 7.9, 10 mM KCl, 1.5 mM MgCl_2_, 0.5 mM PMSF, 0.1 mM EDTA, 2 μg/mL leupeptin, 2 μg/mL antipain, 30 mM NaF, 3 mM sodium orthovanadate, 20 mM sodium pyrophosphate and 5 mM BG-P) and incubated on ice for 15 min. NP-40 was added, and the samples were homogenized and centrifuged for the 30 s at 13,000*g* at 4 °C. Supernatants were used for Western blotting assay and pellets were resuspended in extraction buffer (1.5 mM MgCl_2_, 20 mM HEPES, pH 7.9, 25% glycerol, 300 mM NaCl, 0.5 mM PMSF, 0.25 mM EDTA, 2 μg/mL leupeptin, 2 μg/mL antipain, 3 mM sodium orthovanadate, 30 mM NaF, 20 mM sodium pyrophosphate and 5 mM BG-P) and kept on ice for 20 min. Samples were centrifuged for 20 min at 13,000*g* at 4 °C and supernatants were aliquoted as nuclear extract used for EMSA assay. Protein concentration was determined using the Bradford protein reagent (BioRad).

### Electrophoretic mobility shift assay (EMSA)

Nuclear extracts from control or treated cells were prepared as previously described^22^. A double-stranded oligonucleotide containing the NF-κB consensus sequence from Promega (5′-AGTTGAGGGGACTTTCCCAGGC-3′) was end labeled using T4 polynucleotide kinase (Promega) in the presence of γ-32P dATP. Nuclear extracts (2.5 μg) were incubated with a ^32^P-labeled NF-κB probe. The binding reaction was performed at room temperature for 30 min in a reaction buffer containing 50 mM Tris–HCl pH 7.5, 250 mM NaCl, 5 mM MgCl_2_, 2.5 mM EDTA, 20% glycerol, 0.25 μg/μL of poly (dI–dC) and 2.5 mM dithiothreitol. DNA protein complexes were separated by electrophoresis through a 6% acrylamide:bis-acrylamide (37.5:1) gel in TBE (45 mM Tris, 45 mM boric acid, 0.5 mM EDTA) for 2 h at 150 V. Gels were vacuum dried for 1 h at 80 °C and exposed to X-ray film at − 80 °C. For competition assays, the nuclear extract was incubated with a specific competitor (unlabelled double-stranded NF-κB consensus oligonucleotide) or a non-specific competitor (unlabelled transcription initiation factor IID [TFIID]). For the supershift assay, antibodies against subunits of NF-κB (p50 and p65, 1:20) (Santa Cruz Biotechnology) were added to the binding reactions. Autoradiographs were visualized using a photo documentation system DP-001-FDC and quantified with ImageJ (NIH) software 70.

### Western Blotting

Electrophoresis was performed using 10% polyacrylamide gel and the Bio-Rad mini-Protean III apparatus (Bio-Rad, Hercules, CA, U.S.A.). In brief, the proteins present in cytosolic and nuclear fractions were size-separated in 10% SDS-PAGE (90 V). The immunoblotting was performed as described previously^27^. The proteins were blotted onto a nitrocellulose membrane (Bio-Rad, Hercules, CA, U.S.A.) and incubated with the specific antibody: RelA (p65) (1:1000, sc-0372; Santa Cruz Biotechnology, Santa Cruz, CA, USA), and β-actin (1:2000 (58169; Cell Signaling, U.S.A.) and after with secondary antibody (Rabbit). Proteins recognized by antibodies were revealed by an electrochemiluminescence (ECL) technique, following the Manufacturer's instructions (Amersham Biosciences, Amersham, U.K.). To standardize and quantify the immunoblots, we used the photo documentation system DP-001-FDC (VilberLourmat, Torcy, France) and N.I.H. ImageJ software (http://rsb.info.nih.gov/ij). Several exposure times were analyzed to ensure the linearity of the band intensities.

### Statistical analysis

Results are expressed as mean ± S.E.M. of the indicated number of experiments. Statistical comparisons for α-Klotho-induced changes in cytokines, Western blotting, and cell viability were performed by one-way analysis of variance (ANOVA), followed by the Tukey post-test. All analyses were performed using a Prism 9 software package (GraphPad Software, San Diego, CA, U.S.A.). *P*-values < 0.05 were considered to reflect a statistically significant difference.

### Ethical approval and consent to participate

Both cell culture preparation (glial cells and neurons) was conducted under The Ethical Principle in Animal Research adopted by the Brazilian College of Animal Experimentation (CONCEA) and were approved by the Ethical Committee for Animal Research (CEEA) of the Biomedical Sciences Institute of the Universidade de São Paulo, São Paulo, São Paulo State, Brazil. The protocol was registered under number 12/2016 CEEA of animals used for experimentation.

## Results

### Effect of α-Klotho and LPS treatments on cell viability and cytotoxicity of primary glia culture

**Figure 1:**
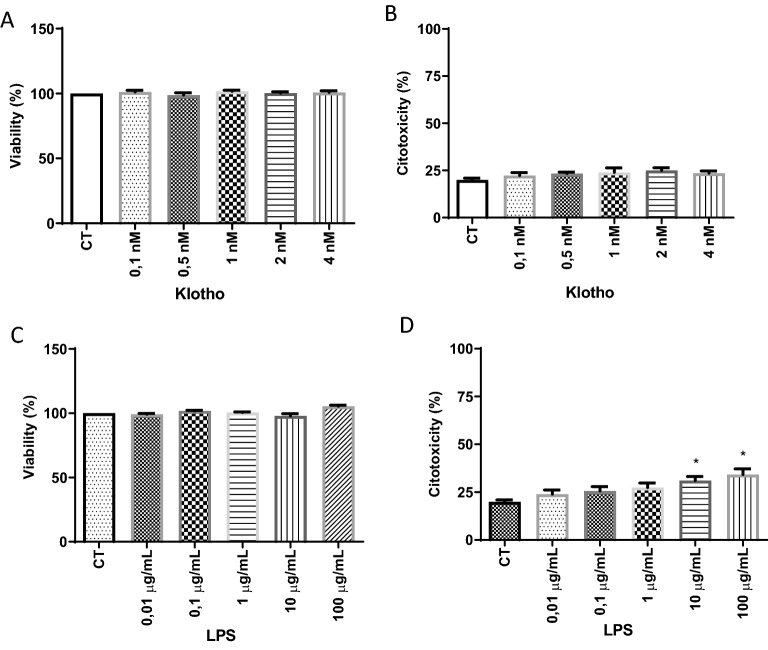
Effect of α-Klotho (**A**,**B**) and LPS (**C**,**D**) treatment on cell viability (**A**,**C**) and cytotoxicity (**B**,**D**) of mouse glial cells. The primary culture of glial cells was subjected to 24-h treatment with LPS in different concentrations. Cell viability and cytotoxicity were assessed by MTT and LDH assays, respectively. One-way ANOVA analysis, followed by Tukey's post-test, *p < 0.05. Results are presented as mean ± SEM of 7 independent experiments.

The different α-Klotho concentrations (0.1–4 nM) did not alter cell viability or show toxicity to glial cells in both MTT and LDH assays (Fig. 1A,B). Regarding the LPS challenge, concentrations between 0.01 and 100 ug/mL caused no effect in the MTT assay, but in the LDH assay, only concentrations of 10 and 100 ug/mL showed toxicity to glial cells when compared to the control group (Fig. 1C,D). Based on these data, the concentration of 1 µg/mL of LPS was chosen for the subsequent experiments.

### Effect of α-Klotho on LPS-induced cytokines secretion in primary mouse glia culture

**Figure 2:**
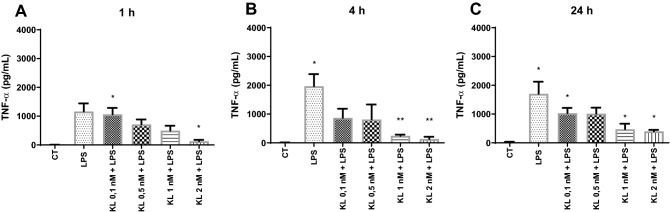
Effect of α-Klotho on LPS-induced TNF-α secretion in mouse glial cells. The glial culture was pre-treated with serum-free medium (control) or with α-Klotho protein in different concentrations (0.1, 0.5, 1, and 2 nM) and times 1 (**A**), 4 (**B**), and 24 (**C**) hours, and then challenged with 1 µg/mL/mL LPS for 8 h. The supernatant was collected to measure TNF-α levels. One-way ANOVA analysis, followed by Tukey's post-test, *p < 0.05, **p < 0.01. Results are presented as mean ± SEM of 7 independent experiments.

After analyzing cell viability and toxicity of different concentrations of α-Klotho in primary glia culture, time-course and concentration of α-Klotho effects on LPS-induced TNF-α secretion was evaluated. This cytokine was chosen based on previous studies in glial cells showing an increase of this cytokine after LPS treatment^28^. Thus, concentrations ranging from 0.1 to 2 nM of α-Klotho and different time points of treatment (1, 4 and 24 h) were used, followed by a LPS challenge (1 µg /mL for 8 h) and TNF-α levels were determined by ELISA. Data showed that LPS -induced an increase in TNF-α levels compared to the control group, and this effect was blocked by α-Klotho protein pretreatment at concentrations of 2 nM for 1 h, and 1–2 nM for 4 and 24 h (Fig. 2A–C).

**Figure 3:**
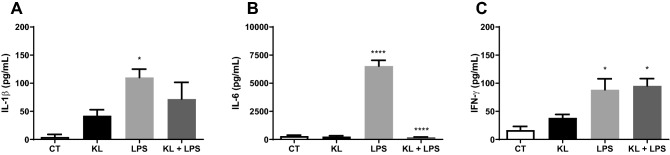
Effect of α-Klotho on IL-1β, IL-6, and IFN-γ levels in mouse cell glia challenged with LPS. The glial culture was pretreated with serum-free medium (control) or with 1 nM α-Klotho for 24 h, and then challenged with 1 µg/mL/mL LPS for 8 h. The supernatant was collected to measure the levels of IL-1β (**A**), IL-6 (**B**), and IFN-γ (**C**). One-way ANOVA analysis, followed by Tukey's post-test, *p < 0.05, **p < 0.01, **** < 0.0001. Results are presented as mean ± SEM of 7 independent experiments.

Based on these data, the concentration of 1 nM for 24 h was chosen for evaluating the influence of α-Klotho on LPS (1 µg/mL for 8 h) induced change in proinflammatory cytokines. In addition to TNF-α, LPS increased the production and secretion of other pro-inflammatory cytokines, such as IL-1β, IL6, and IFN-γ (Fig. 3A–C). However, α-Klotho treatment (1 nM for 24 h) only reversed the LPS -induced increase of IL-6 levels (Fig. 3B) but not of IL-1β and IFN-γ (Fig. 3A,C).

### Effect of α-Klotho on LPS-induced NF-κB activation in astrocytes primary mouse glia culture

**Figure 4:**
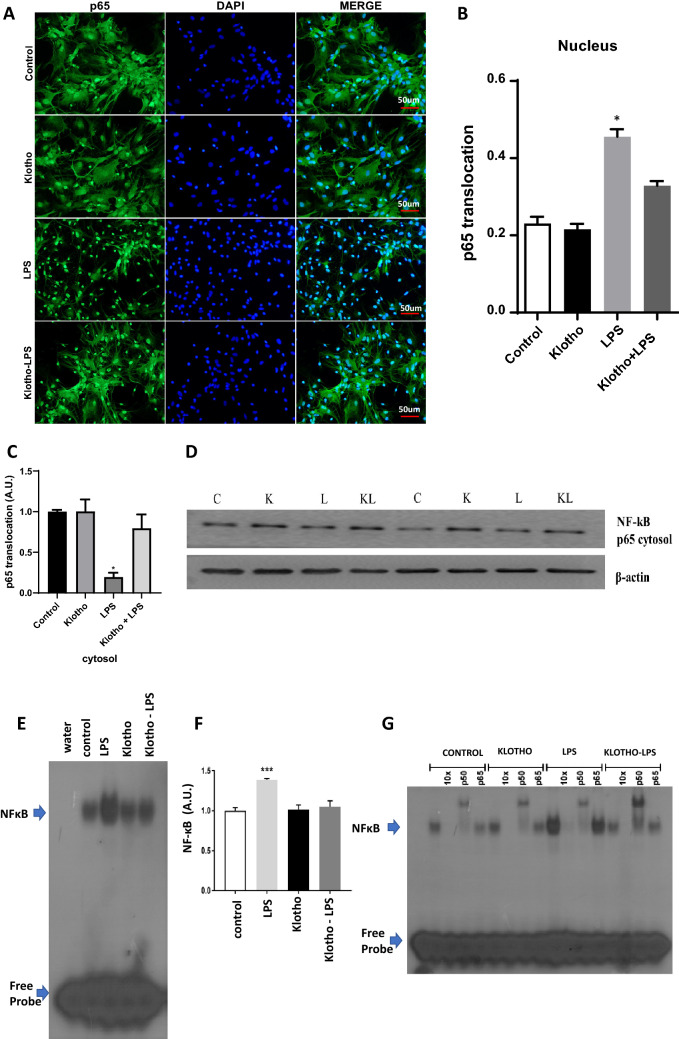
α-Klotho rescue the NF-κB activation induced by LPS in the nuclear fraction. (**A**) Purified astrocytes from cortical glial cells were treated for 24 h with vehicle (control) (PBS) or 1 nM α-Klotho. Inflammatory stimulation with LPS 1 μg/ml was then performed. (**B**) The RelA (p65)-positive nuclei were counted and divided by the total number of nuclei, and the graph shows the comparison between the LPS group and for α-Klotho + LPS group expressed by the ratio in arbitrary units of RelA (p65) translocated to the nucleus over the total amount of RelA (p65) (n = 5). One-way ANOVA analysis, followed by Tukey's post-test, **p < 0,01; ***p < 0,001; ****p < 0,0001. (**C,D**) Effect of α-Klotho on p65 subunit NFkB translocation in astrocytes cells. Cytosol (20 mg) proteins were extracted from primary cultured cells: (**C**) Representative Western blotting autoradiographs of RELA (p65) cytosolic and β-actin; (**D**) Densitometric analysis (arbitrary units, A.U.) of p65 cytolosic/β-actin ratios of groups presented in the panel (n = 5). One-way ANOVA analysis, followed by Tukey's post-test, **p < 0,01; ***p < 0,001; ****p < 0,0001. (E) Nuclear fraction was used to perform the EMSA assay to measure NF-kB activity. (**F**) Densitometric analysis comparing NF-κB activity of control, α-Klotho, LPS, and α-Klotho–LPS groups (n = 5). One-way ANOVA analysis, followed by Tukey's post-test,***p < 0,0001. (**G**) A super-shift was also performed to show which NF-κB subunits are involved in this phenomenon.

To evaluate the influence of α-Klotho on NF-κB signaling we performed experiments in astrocyte primary culture based on previous evidence that secreted neuronal α-Klotho modulates astrocytic metabolic activity^15^. For immunofluorescence experiments, we used staining of RelA (p65), which is an NF-kB subunit that is activated during LPS-induced inflammation (Fig. 4). Pre-treatment of cells with concentrations of 1 nM α-Klotho for 24 h, followed by challenge with LPS at 4 h (Fig. 4A, B) confirmed increased RelA nuclear translocation by LPS vs*.* control. RelA nuclear translocation induced by LPS was completely reverted by recombinant α-Klotho treatment (Fig. 4A, B). For a quantitative assessment, cells extracted from cytosolic and nuclear fractions were used to evaluate the RelA (p65) content in each compartment by Western Blotting (Fig. 4C). Results confirmed immunofluorescence data as the RelA (p65) subunit translocation subunit induced by LPS is inhibited by recombinant α-Klotho (Fig. 4C). Finally, these groups were submitted to an EMSA assay to more precisely detect whether the NF-kB that translocated to the nucleus was active and bound to its specific sequence in DNA (Fig. 4D, E). EMSA data confirmed both previous data as the binding of the nuclear extract to the 32P-labeled probe was much higher in LPS-treated astrocytes, and pre-treatment with α-Klotho was able to inhibit the activation of this transcription factor. A super-shift assay was also performed (Fig. 4F) to clarify that NF-kB subunits are involved in this activation. Data confirmed that RelA (p65) and p50 subunits are involved, which typically occurs following activation by LPS^29^.

### Effects of α-Klotho on GCM -induced cytotoxicity of mouse primary neuronal culture

**Figure 5:**
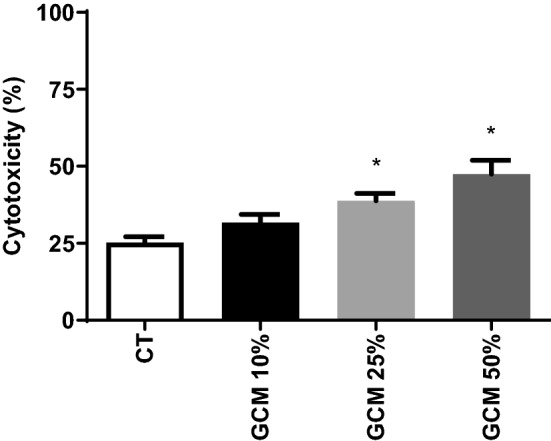
Effects of GCM -induced cytotoxicity of mouse neurons. The primary embryonic culture of neurons was subjected to 24-h treatment with GCM in different concentrations (25% and 50%). Cytotoxicity was assessed by the LDH assay. One-way ANOVA analysis, followed by Tukey's post-test, *p < 0.05. Results are presented as mean ± SEM of 5 independent experiments.

Primary glia culture was pretreated with serum-free medium (GCM) or with 1 nM α-Klotho (GCM-KL) for 24 h and then challenged with 1 µg/mL LPS for 24 h. Initially, it was necessary to determine the amount of GCM coming from the LPS-challenged glial cell (GCM group) which caused neuronal death. Thus, we replaced 10, 25, and 50% of the cultured medium of the neuronal culture with GCM. The results showed that when the concentration of 25 and 50% of the neuronal culture medium was switched to GCM, there was an increase in neuronal toxicity when compared to the control group (Fig. 5).

**Figure 6:**
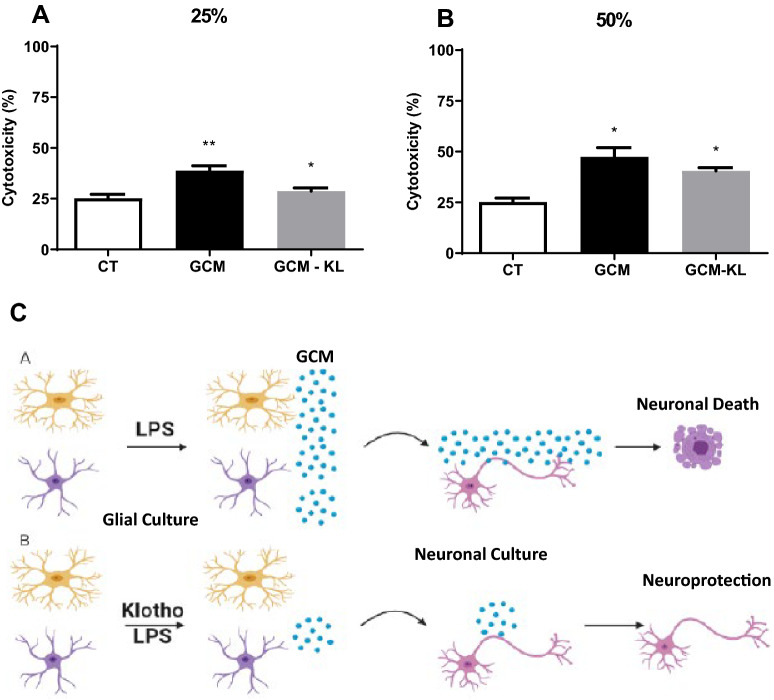
Effects of GCM and GCM-KL -induced cytotoxicity of primary cortical mouse neurons. The primary embryonic culture of neurons was submitted to 24-h treatment with GCM or GCM-KL in different concentrations, 25% (**A**) and 50% (**B**). Cytotoxicity was assessed by the LDH assay. One-way ANOVA analysis, followed by Tukey's post-test, *p < 0.05, **p < 0.01. Results are presented as mean ± SEM of 5 independent experiments. (**C**) Representative schedule of the anti-inflammatory and neuroprotective effect of α-Klotho protein. LPS induces GCM to produce pro-inflammatory mediators that can lead to neuronal death (**A**). α-Klotho protein decreases the production of pro-inflammatory mediators induced by LPS in GCM (**B**), and it can have a protective effect on neurons from the neurotoxic effects of LPS induced by GCM. The figure was “Created with BioRender.com—Agreement number *FH248VCXX8 to Scientific Reports”.*

**Figure 7:**
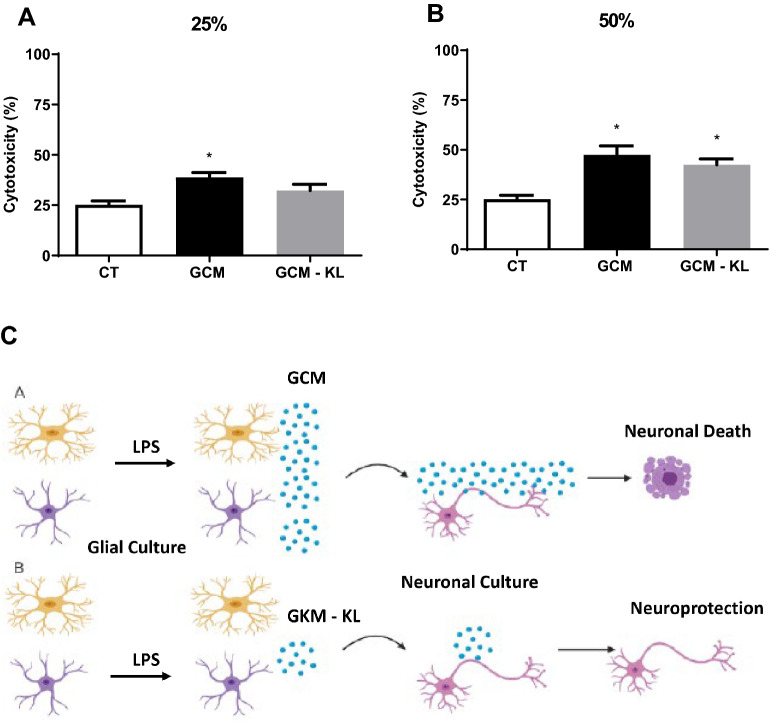
Effects of Klotho on GCM -induced cytotoxicity of primary cortical mouse neurons. The primary embryonic culture of neurons was submitted to 24-h treatment with GCM in different concentrations, 25% (**A**) and 50% (**B**) in the presence or absence of KL. Cytotoxicity was assessed by the LDH assay. One-way ANOVA analysis, followed by Tukey's post-test, *p < 0.05, Results are presented as mean ± SEM of 5 independent experiments. (**C**) Representative schedule of the neuroprotective effect of α-Klotho protein on GMP-induced cytotoxicity of neurons. LPS induces GCM to produce pro-inflammatory mediators that can lead to neuronal death (**A**). α-Klotho protein decreases the production of pro-inflammatory mediators induced by LPS in GCM (**B**), and it can have a protective effect on neurons from the neurotoxic effects of LPS induced by GCM. The figure was “Created with BioRender.com—Agreement number *FH248VCXX8 to Scientific Reports*”.

After determining the concentration of GCM that induced neurotoxicity, we investigated whether the GCM from α-Klotho pretreated glial culture (1 nM) and stimulated with LPS (GCM-KL) would be able to decrease neuronal death when compared to GCM at both 25 and 50% concentrations (Fig. 6). The results showed that 25% of GCM-KL reversed the increase in neuronal death caused by GCM (Fig. 6A), but α-Klotho pretreatment was not able to induce any effect to a high concentration (50% of GCM-KL) (Fig. 6B). We also evaluate the neuroprotective effect of KL on GCM -induced neurotoxicity of primary cortical mouse neurons. The primary embryonic culture of neurons was submitted to 24-h treatment with GCM (stimulated or not with LPS) in different concentrations, 25% (A) and 50% (B) in the presence or absence of KL. Cytotoxicity was assessed by the LDH assay (Fig. 7). The results confirmed KL neuroprotective action since the presence of this protein in GCM -challenge incubation to neurons was able to revert the increase in neuronal death caused by 25% of GCM (Fig. 7A), but not at 50% of GCM (Fig. 7B).

## Discussion

Neuroinflammation is a key feature of the aging process, neurodegenerative diseases, and injuries that affect the CNS, and is characterized by the activation of microglia and astrocytes. These cells have a fundamental role in the regulation of neuroinflammation. Depending on the nature of their activation, they can lead to the production of pro and/or anti-inflammatory mediators and have both beneficial and detrimental effects on neurons^30,31^. In this work, we sought to evaluate the role of α-Klotho in the modulation of inflammatory processes induced by LPS in glial cells. It is well known that activation by LPS in those cells leads to increased production and secretion of pro-inflammatory cytokines (including TNF-α, IL-6, IL-1, and IL-12). In addition, LPS increases the expression of other pro-inflammatory mediators, such as chemokines (like CXCL8, CCL5, and CCL2), complement system proteins (like C3, C3aR, C5a5, and factor B), and enzymes (like cyclooxygenase type 2 (COX-2) and induced nitric oxide synthase (iNOS)^32,33^. Our data confirmed these data since LPS increased the secretion of pro-inflammatory cytokines, such as TNF-α, IL-6, IL-1β, and IFN-γ.

Pro-inflammatory mediators, such as TNF-α, IL-1β, and IL-6, when produced in excess and/or in a chronic manner by glial cells, can lead to neuronal death^34,35^. Studies have already shown that the application of GCM of microglia and mixed glial culture activated by LPS in neurons leads to neuronal death^28,36–38^. According to these data, our data showed that the application of GCM from LPS-activated GCM in neurons led to a concentration-dependent increase in neuronal death. As the GCM was used, it is not possible to say which molecule is leading to neuronal death. Probably not just one, but a set of mediators, including the proinflammatory cytokines that were elevated after LPS stimulation such as TNF-α, as we previously demonstrated^28^.

The activation of glial cells by LPS leads to an increase in the production of proinflammatory mediators in glial cells and these mediators are capable of inducing neuronal death^39^. Therefore, we investigated whether α-Klotho would be able to decrease the effects induced by LPS in glial cells since the protective and anti-inflammatory activity of α-Klotho protein has already been seen in the renal, vascular and pulmonary systems^40^. However, the protective effect of α-Klotho protein in neuroinflammation has been poorly studied. α-Klotho has been shown to decrease NF-κB activation and reduce the production of pro-inflammatory cytokines, such as TNF-α, IL-6, IL-8, and IL-1β, in vivo and in vitro in models of cardiac inflammation^41,42^, kidney disease^43^ and lung disease^44,45^. For example, in human kidney embryonic cells (HEK293), Zhao et al*.* demonstrated that pretreatment with 200 pM of α-Klotho for 45 min was able to decrease NF-κB activation by approximately 70% and reduce the expression of IL-8, MCP-1, RANTES, and IL-6 after the addition of TNF-α^43^. In HUVECs cells, the same pretreatment for 6 h has been shown to decrease NF-κB activation and expression of adhesion molecules, intercellular adhesion molecule 1 (ICAM-1), and adhesion molecule of vascular cell 1 (VCAM-1), induced by TNF-α^46^.

Recent studies have demonstrated the protective and anti-inflammatory effect of α-Klotho in the CNS. α-Klotho overexpression in the mouse choroid plexus improved behavioral deficit and increased the number of live neurons after cerebral hypoperfusion, accompanied by a decrease in translocation p65 from the cytoplasm to the nucleus, production of proinflammatory cytokines, and activation of astrocytes and microglia^47^. A recently published study showed that α-Klotho's systemic overexpression in an experimental model of amyotrophic sclerosis (mouse transgenic for superoxide dismutase 1) led to later onset and progression of the disease and increased survival of these animals^48^. Still, it was observed that α-Klotho decreased the expression of inflammatory markers and prevented neuronal death. In addition, a reduction in the secretion of TNF-α, IL-6, and the expression of iNOS and COX-2 induced by LPS/IFN-γ in mouse microglia culture that overexpressed α-Klotho^48^.

In our study, α-Klotho was able to decrease the secretion of pro-inflammatory cytokines, TNF-α and IL-6. Pretreatment with 1 nM α-Klotho for 4 and 24 h and 2 nM for 1, 4, and 24 h decreased LPS-induced TNF-α secretion in glial cells. Also, pretreatment with 1 nM α-Klotho for 24 h reversed the increase in IL-6 secretion induced by LPS.

Interestingly, experiments in astrocytes purified glial cells reinforce the anti-inflammatory action of α-Klotho as pretreatment with this recombinant protein significantly reduces nuclear translocation of Rel A (p65) subunit of NF-κB induced by LPS as revealed by immunofluorescence and Westering blotting experiments. In addition, EMSA data confirmed that α-Klotho was able to revert the p65/p50 NF-κB activation induced by LPS suggesting a putative neuroprotective action of this protein. The present data reinforce the importance of understanding the roles of astrocytes and microglia in neurodegenerative diseases to develop effective therapies for neurodegenerative diseases^49^. Considering that insulin and glutamate-induced neuronal secreted α-Klotho play an important role in brain metabolism and neuroinflammation by modulating neuron-astrocyte coupling^15^ it would be important to consider it an important player in the complex activated glial cells during degenerative diseases.

When the conditioned medium of glial cells was preincubated with α-Klotho (1 nM) for 24 h before they were challenged with 1 µg/mL LPS for 8 h (GCM-KL), the neuronal death caused by the GCM of glial cells treated only with LPS was rescued in a lower concentration of GCM (25%), reinforcing, the therapeutic potential of α-Klotho in the CNS shown in other studies. In addition, KL (1 nM) incubation for 24 h together with GCM of glial cells treated with LPS for 8 h completely revert the neuronal to a lower concentration of GCM (25%), confirming the neuroprotective action of α-Klotho.

It is unclear which mediators were leading to neuronal death, as well as if α-Klotho is the only mediator involved in the neuroprotective effect. It may have been due to the decrease in TNF-α and IL-6 levels, which were observed in this study, as well as the decrease in other inflammatory mediators not evaluated or by inducing an adaptive response linked to an increase in protective factors, such as BDNF, or GNFT. In addition, α-Klotho may have had this protective effect due to the secretion of anti-inflammatory mediators and/or neuroprotective factors. Studies that have demonstrated an anti-inflammatory effect of α-Klotho observed a decrease in NF-κB activation^43,46^. This modulation of NF-κB activation was also observed in the CNS^47^. Our data confirmed that at least part of the mechanism by which α-Klotho could be leading to this anti-inflammatory and neuroprotective effect is mediated by NF-κB. The pretreatment with α-Klotho led to a decrease in the activation of NF-κB in astrocytes and, consequently, resulted in a decrease in the production and secretion of proinflammatory cytokines. Interestingly, previous studies from our laboratory showed that α-Klotho has a strong influence on the astrocytic metabolism stimulating aerobic glycolysis and lactate release mediated by FGFR1 and Erk1/2 activation^15^. The ability of α-Klotho to modulate the FGFR and NF-κB signaling in astrocytes is consistent with results in other cell types^50^ and suggests that in CNS α-Klotho can modulate neuroinflammation by neuron-glia coupling action.

## Conclusions

In conclusion, our work demonstrated for the first time in primary cortical neuronal culture an anti-inflammatory and neuroprotective effect of the α-Klotho protein as the cytotoxicity of GCM could be rescued by α-Klotho pre-treatment (GCM-KL), as well as, by α-Klotho + GCM challenged with LPS -induced neurotoxicity. This neuroprotective α-Klotho effect is at least partially mediated by astrocyte/glia NF-κB modulation of neuroinflammation. These data suggest that α-Klotho can act not just in the metabolic coupling between neurons and astrocytes^15^, but it is also an important player in modulating glia neuroinflammation which is important to integrate insulin and glutamate action in neurons. Thus, α-Klotho's therapeutic potential is evidenced in pathological processes that have a neuroinflammatory component.

## Supplementary Information

Supplementary Information.

## Data Availability

"The datasets used and/or analysed during the current study available from the corresponding author on reasonable request".

## Abbreviations

ADAM10: Metalloproteinase 10
ADAM17: Metalloproteinase 17
BACE1: β-Secretase 1
BDNF: Brain-derived neurotrophic factor
CNS: Central Nervous System
COX-2: Cyclooxygenase-2
CSF: Cerebral Spinal Fluid
CXCL: C-X-C Motif Chemokine Ligand
DMEM: Dulbecco´s Modified Eagle Medium
EMSA: Electrophoretic mobility shift assay
Erk1/2: Extracellular signal-regulated protein kinase
FBS: Fetal bovine serum
FGF: Fibroblast Growth Factor
GCM: Glia conditioned medium
GCM-KL: GCM pretreated with α- α-Klotho followed by LPS
GFAP: Glial fibrillary acidic protein
IFN-γ: Interferon-γ
IL-1β: Interleukin 1 beta
IL-6: Interleukin-6
iNOS: Inducible nitric oxide synthase
LDH: Lactate dehydrogenase
LPS: Lipopolysaccharides
MTT: 3-(4,5-Dimethylthiazol-2-yl)-2,5-diphenyltetrazolium bromide)
NF-κB: Nuclear factor kappa B
p50: NF-κB-subunit
RelA (p65): NF-κB- subunit
TLR4: Toll-like receptor 4
TNF-α: Tumor necrosis factor-alpha
α-Klotho: Klotho
