# Supplementary material for: Neuroprotective action of α-Klotho against LPS-activated glia conditioned medium in primary neuronal culture
Source: Sci Rep. 2022 Nov 7;12:18884. doi: 10.1038/s41598-022-21132-4 (PMC9640694; doi:10.1038/s41598-022-21132-4)

Photomicrographs of laser confocal microscopy of the primary glial culture cells submitted to immunofluorescence with anti-GFAP, anti-MAP2, and anti-CD68 antibody and nuclear marker DAPI (A and D). The pattern of immunoreactivity to GFAP, astrocyte marker (B). Absence of neurons in glial culture Overlapping images of A and B, showing the presence of astrocytes in the glial culture (C). Immunoreactivity to CD68 is present in the glial culture (E). (F) Overlapping images of D and E, where we can observe a sample of the glial population, characterized by the presence of astrocytes and microglia.

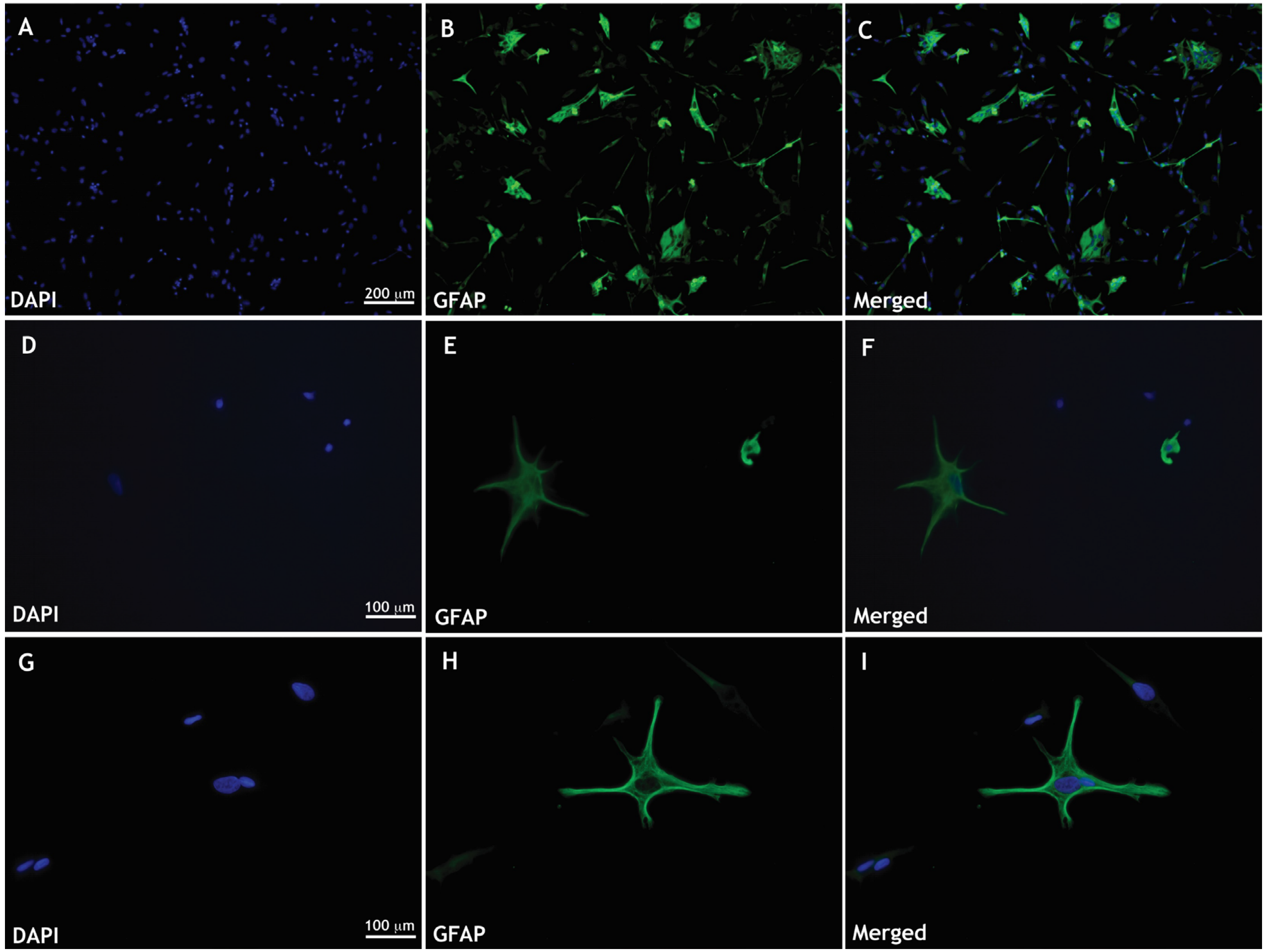

Photomicrographs of laser confocal microscopy of the primary neuronal culture cells submitted to immunofluorescence with Pan-Neuronal and NeuN antibodies and nuclear marker DAPI.
Overlapping images of Pan-Neuronal and NeuN, showing the presence of neurons in the culture.

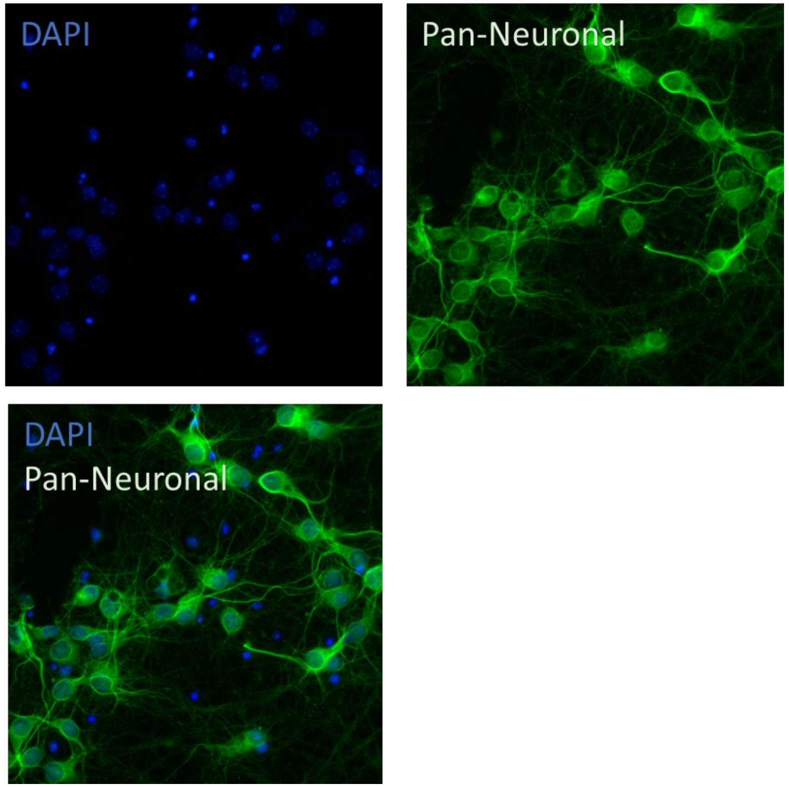

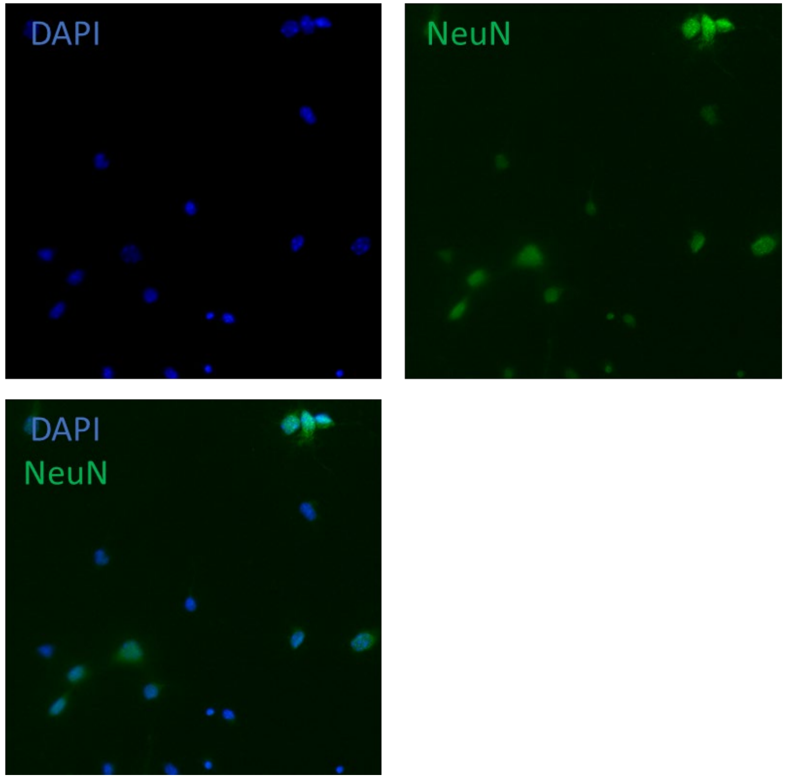

EMSA assay to measure NF-kB activity

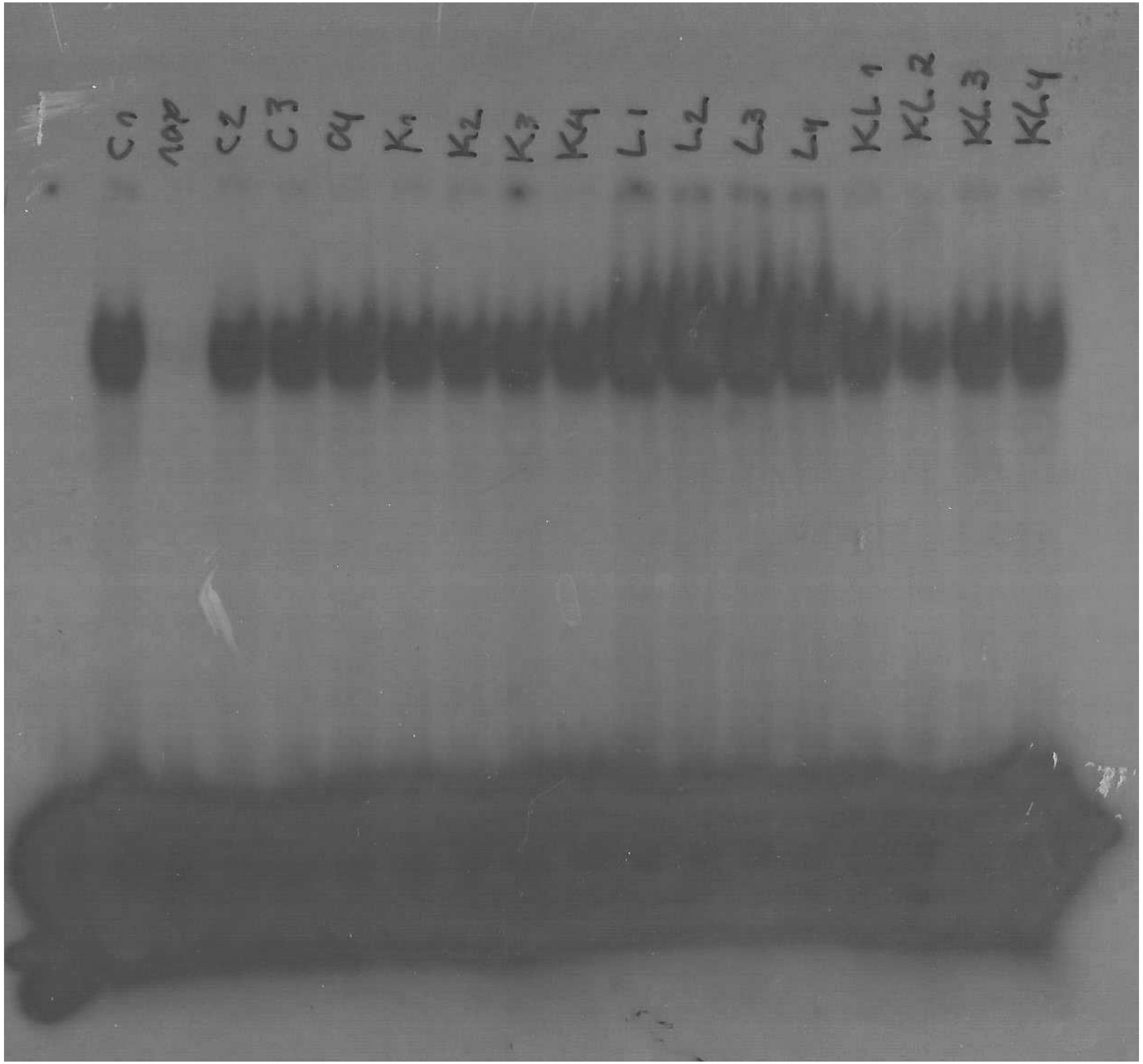

NF-kB (p65/p50)

Free Probe

Groups:
C = Control
K = Klotho
L = LPS
KL = Klotho + LPS

Super-shit EMSA assay to measure NF-kB activity

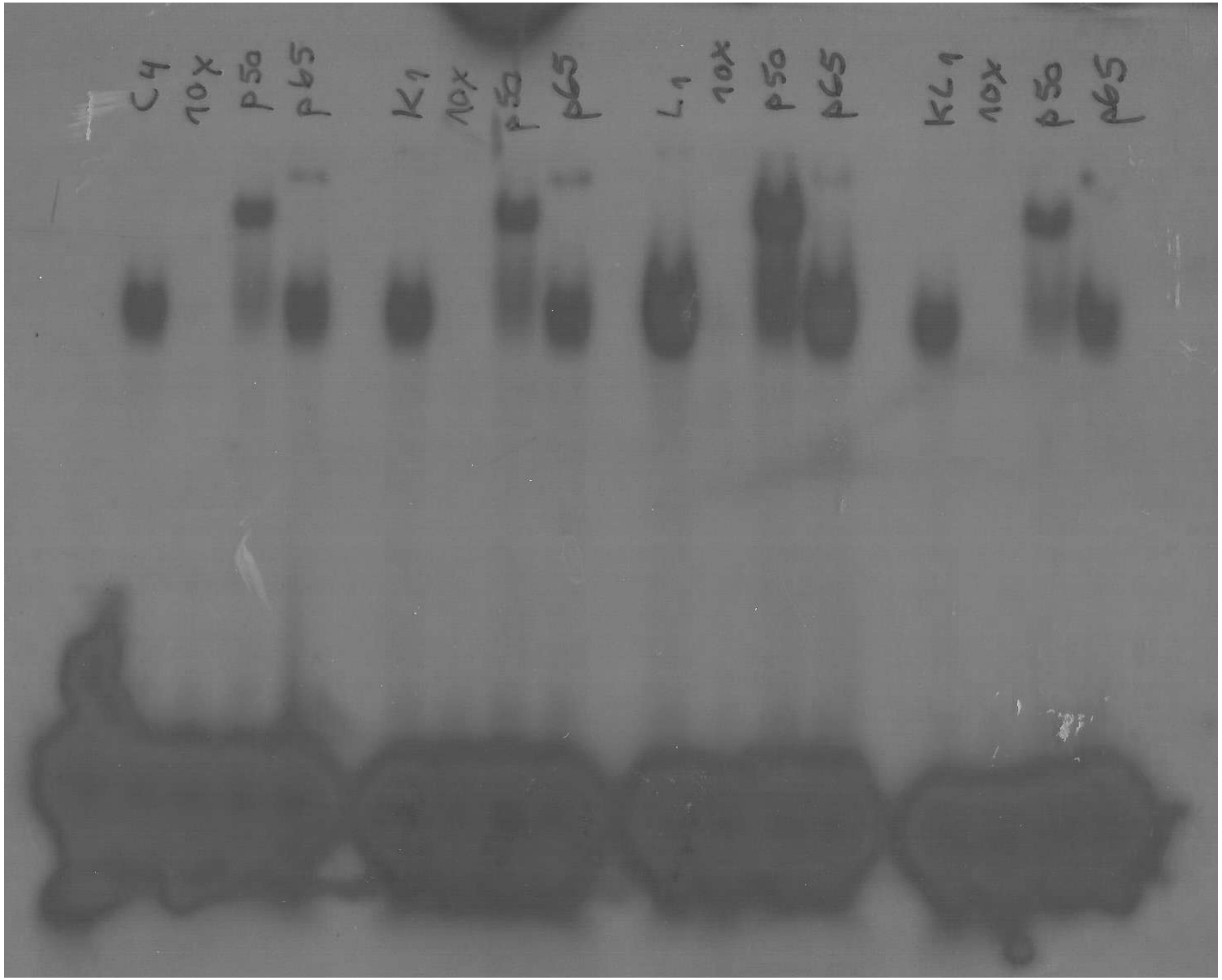

Groups:
C = Control
K = Klotho
L = LPS
KL = Klotho + LPS

NF-kB

Free Probe

Western blotting autoradiographs of RELA (p65) cytosolic and β-actin

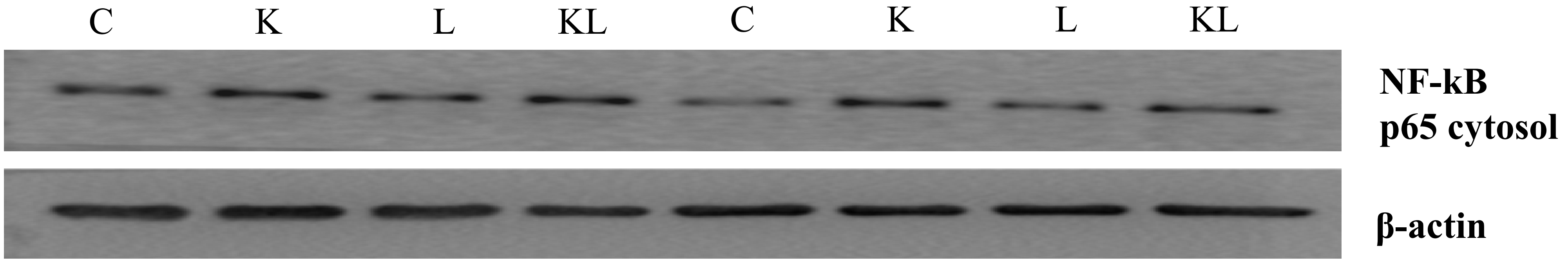

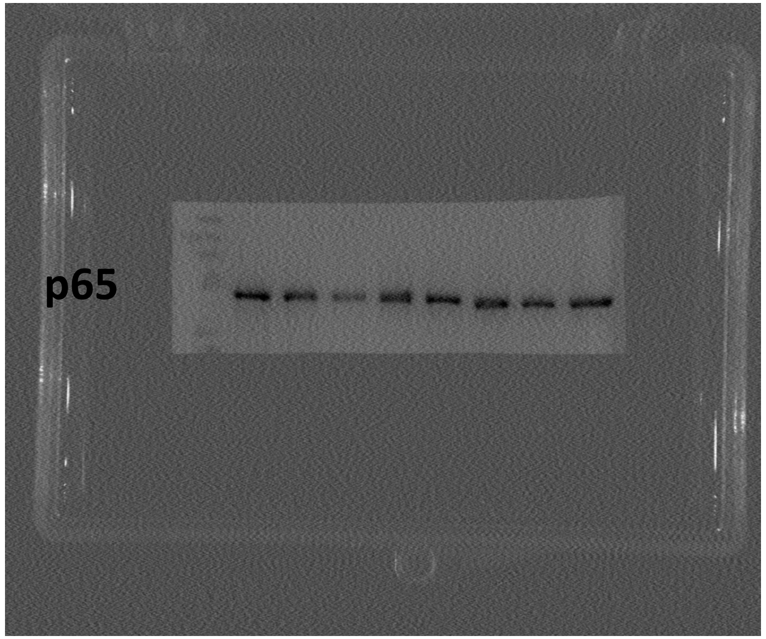

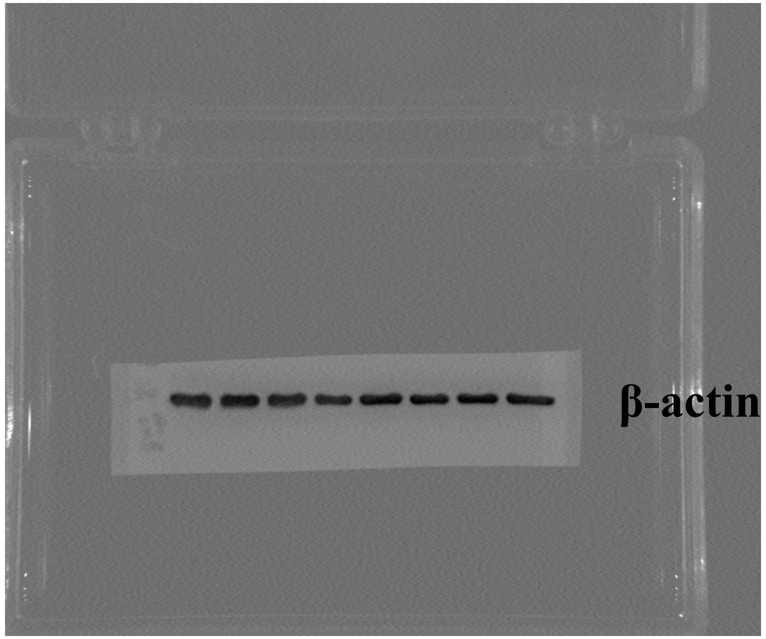

Western blotting autoradiographs of RELA (p65) cytosolic and β-actin

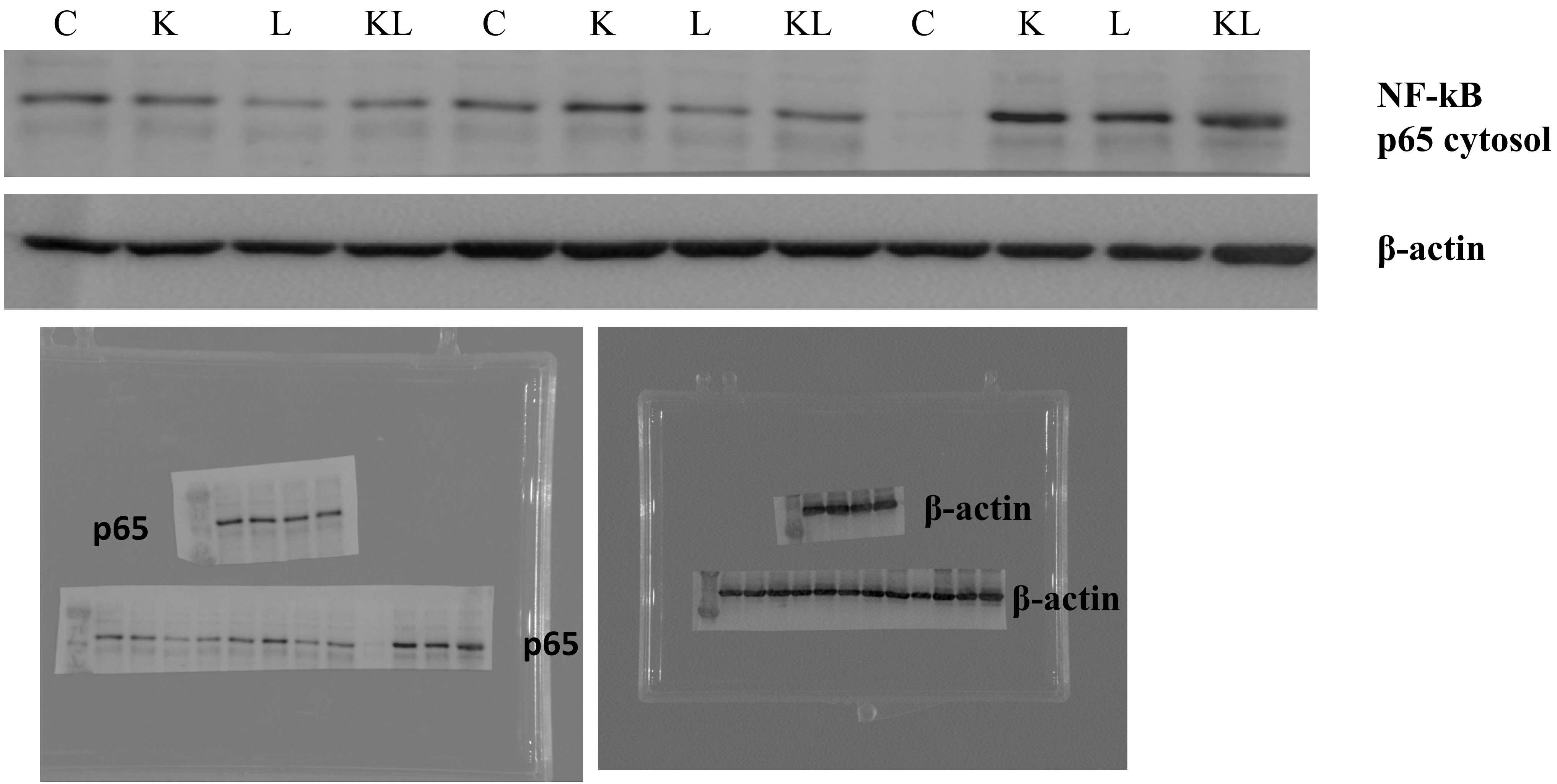

Supplement: Supplementary file 1 — Supplementary Information. [file 41598_2022_21132_MOESM1_ESM.pdf]
